# Age-Related Patterns of Female Suicide in Türkiye: A 15-Year Nationwide Analysis of Reported Reasons and Methods

**DOI:** 10.3390/bs16040490

**Published:** 2026-03-26

**Authors:** Gökmen Karabağ, Volkan Zeybek, Mehmet Sunay Yavuz

**Affiliations:** Department of Forensic Medicine, Faculty of Medicine, Manisa Celal Bayar University, 45140 Manisa, Türkiye; volkan.zeybek@cbu.edu.tr (V.Z.); sunay.yavuz@cbu.edu.tr (M.S.Y.)

**Keywords:** female suicide, Türkiye, age groups, suicide causes, suicide methods, hanging, chronic illness, national statistics, gender-based analysis

## Abstract

Suicide is a major public health problem worldwide, and its reported reasons and methods show marked variation by gender and age. Although suicide rates are generally higher among men, suicides among women demonstrate distinct sociodemographic and age-related patterns that remain insufficiently explored. In Türkiye, national suicide statistics are available; however, nationwide, age-stratified analyses focusing exclusively on women are limited. This study aimed to investigate long-term trends, age-related differences in reported reasons and methods of suicide among women in Türkiye, and to provide insights relevant to age- and gender-sensitive prevention strategies. This retrospective, nationwide descriptive study analysed female suicide data obtained from the Turkish Statistical Institute between 2009 and 2023. A total of 12,868 female suicide cases were included (mean age 36.5 ± 19.3 years). Data were evaluated according to year, age group, marital status, educational level, suicide cause, and suicide method. Causes and methods were classified based on official administrative categories. Descriptive statistics were calculated, and associations between age groups and suicide causes and methods were assessed using Pearson’s chi-square test. During the 15-year study period, 12,868 women died by suicide in Türkiye. The annual suicide rate ranged from 1.81 to 2.46 per 100,000 population, with the lowest rate observed in 2017 and the highest in 2022. Among all age groups, the most frequent cause of suicide was illness, especially in women aged 45 and older. The proportion of suicides due to illness was 13.9% in the 15–24 age group, 24.6% in 25–34, 41.0% in 45–54, and 42.3% in 55–64 (*p* < 0.001). Emotional and relationship-related causes were more prevalent among younger women, particularly in the 15–24 age group (4.8%), but declined significantly with age (*p* < 0.001). Economic hardship was the least cited cause overall, especially among women under 35 (*p* < 0.001). Regarding methods of suicide, hanging was the most common method in all age groups and increased with age—35.8% in 15–24, 55.1% in 45–54, and 63.5% in 75+ age group (*p* < 0.001). The use of chemical substances peaked in the 15–24 age group (12.4%) and declined in older women (5.8% in 75+). Firearm use showed a significant inverse relationship with age, from 24.6% in those under 15 to 0.8% in women aged 75 and over (*p* < 0.001). These age-related differences in both the causes and methods of suicide were statistically significant (*p* < 0.001). Female suicide in Türkiye exhibits pronounced age-dependent differences in both causes and methods. Illness-related suicides and hanging predominate in older age groups, while younger women show a more diverse pattern of reported reasons and methods. The high prevalence of nonspecific classifications highlights limitations in current suicide reporting systems. These findings underscore the need for improved suicide classification, enhanced surveillance, and age- and gender-sensitive prevention strategies tailored to women across the lifespan.

## 1. Introduction

Suicide is a critical global public health issue and ranks among the leading causes of preventable death worldwide. According to the World Health Organization (WHO), more than 700,000 individuals die by suicide each year, and it is estimated that for every suicide, there are more than 20 suicide attempts globally (Bertuccio et al., 2024). Suicide is not only a health-related problem but also a social phenomenon influenced by a wide range of psychological, socioeconomic, cultural, and demographic factors (Holden & Dwyer, 2023; Rotejanaprasert et al., 2025).

In Türkiye, suicide continues to represent a growing concern for both public health institutions and society at large. While overall suicide rates in the country are lower than global averages, gender-specific trends reveal troubling dynamics. Particularly, female suicides—often overshadowed by higher male suicide rates—have distinctive patterns in terms of age distribution, educational level, marital status, suicide method, and underlying motives, such as family discord or emotional relationships (Baziki Cetin & Atilan Fedai, 2024; Santos et al., 2017). These suicides may be indicative of deeper structural inequalities, including gender-based violence, social isolation, economic vulnerability, and limited access to mental health services (Canbaz & Terzi, 2018).

Historically, studies investigating suicide trends in Türkiye have predominantly analysed general population data in which male suicides are numerically predominant, reflecting the well-established epidemiological pattern of higher suicide mortality among men. There is a notable lack of nationwide epidemiological analysis focusing specifically on completed female suicides in Türkiye. Previous studies have primarily examined psychological risk factors or suicidal tendencies among women rather than patterns of completed suicide at the population level (Kilicarslan et al., 2023). Moreover, variations across age groups and reported reasons for suicide—such as illness, family conflict, economic hardship, and emotional distress—have not been sufficiently explored in women (Arqueros et al., 2025; Barakat et al., 2025; Niwhede et al., 2025). Understanding these nuances is essential for designing gender-sensitive prevention strategies.

Suicidal behaviour is widely recognized as a multifactorial phenomenon shaped by complex interactions between biological, psychological, social, and cultural factors. Numerous epidemiological studies have demonstrated that the distribution of suicidal behaviour varies substantially across demographic groups, particularly by gender and age (Asirdizer et al., 2010; de Beurs et al., 2021; Goren et al., 2004). While men generally exhibit higher suicide completion rates worldwide, women often show different patterns in terms of reported reasons, methods used, and psychosocial circumstances surrounding suicide. These gender differences have been attributed to variations in mental health profiles, social roles, coping mechanisms, and exposure to stressors such as interpersonal conflict or caregiving burdens. Understanding these gender-specific patterns is therefore essential for developing effective prevention strategies tailored to vulnerable populations (Han Yekdes et al., 2025; Niederkrotenthaler et al., 2015; Simion & Jung, 2025; Turecki & Brent, 2016).

Age is another critical determinant influencing suicidal behaviour. Previous research has shown that the relative importance of reported reasons for suicide may shift across the life course (Franck et al., 2020; Graham & Fenelon, 2023). For example, interpersonal conflicts and emotional stressors tend to be more prominent among adolescents and young adults, whereas chronic illness, social isolation, and declining functional capacity may become more relevant in older populations (Kim et al., 2015; Orlins et al., 2020). Despite the importance of these age-related patterns, many national epidemiological studies have analysed suicide trends without detailed age- and gender-stratified approaches. Consequently, there remains a need for nationwide epidemiological analysis focusing specifically on female suicide patterns across different stages of life.

The period between 2009 and 2023 offers valuable longitudinal data for investigating trends in female suicides across Türkiye. National statistics indicate fluctuations in suicide rates over these years, with notable increases in certain regions and among specific demographic groups (Akin & Konar, 2025; Eskin et al., 2021; Han Yekdes et al., 2025; Tuncay & Sarman, 2024). Furthermore, social transformations such as urban migration, increased female participation in the workforce, changes in family structure, and access to education may have affected the psychological well-being of women during this timeframe (Kartal et al., 2022; Kaya et al., 2020; Taktak, 2023).

The broader sociocultural environment in Türkiye further underscores the need for gender-sensitive suicide research. According to the World Economic Forum’s 2023 Global Gender Gap Report, Türkiye ranked 129th out of 146 countries, with a gender equality index score of 0.638, indicating substantial disparities in women’s access to education, economic participation, political representation, and social empowerment (World Economic Forum, 2023; Yılmaz Fındık, 2025). These structural inequalities may contribute to psychosocial vulnerability among women and shape both the motives and contextual factors surrounding female suicides in the country.

This study aimed to examine the sociodemographic characteristics, reported reasons, and methods of suicide among women in Türkiye over a 15-year period. By categorizing data based on age, marital and educational status, and suicide methods, we sought to provide a nationwide epidemiological analysis that may contribute to the development of age- and gender-sensitive suicide prevention strategies. Therefore, the aim of this study was to examine age-related differences of female suicide in Türkiye between 2009 and 2023 using nationwide statistical data.

## 2. Materials and Methods

### 2.1. Study Design and Procedure

Ethical approval was obtained from the Ethics Committee of the Faculty of Medicine, Health Sciences, Manisa Celal Bayar University (Decision Date/No: 3 May 2025–20.478.486/2925). This study was designed as a retrospective descriptive analysis based on nationwide suicide data collected between 2009 and 2023, corresponding to a 15-year observation period. The data concerning female suicides in Türkiye were obtained from the publicly accessible records of the Turkish Statistical Institute (TURKSTAT), which compiles nationwide mortality statistics based on civil registration systems and reports from the Ministry of Interior and the Ministry of Health. These data are publicly available through the official statistical database of TURKSTAT and can be accessed through their online statistical portal. Because the dataset consists of aggregated and anonymized national statistics, no additional institutional permission was required for data access.

Although TURKSTAT employs ICD-10 codes internally when recording mortality statistics, the publicly available suicide dataset used in this study does not include individual ICD-10 codes. Instead, suicides are categorised according to predefined administrative classifications.

In the TURKSTAT database, suicide cases are classified according to reported reasons and methods of suicide. Reported reasons include illness, family conflict, economic hardship, emotional relationship issues or inability to marry the person of choice, and other or unspecified causes. Suicide methods were categorised as hanging, ingestion of toxic substances, jumping from height, use of firearms, and other methods.

In Türkiye, suicide mortality data are compiled through official medico-legal death investigations. When a suicide occurs, information related to the case is collected by law enforcement authorities (police or gendarmerie) using standardised suicide statistics forms. These forms typically include demographic information, suicide method, and the possible reason for suicide based on information obtained during the investigation process. The collected information is subsequently compiled and published by the TURKSTAT as part of national mortality statistics. Similar procedures have been described in previous epidemiological studies using TURKSTAT suicide data (Oner et al., 2015).

The suicide statistics used in this study were obtained directly from the official database of the Turkish Statistical Institute (TURKSTAT), which compiles national mortality data based on medico-legal death investigations. Differences may exist between TURKSTAT statistics and international databases such as Eurostat due to variations in reporting procedures, classification systems, data updates, and the handling of deaths of undetermined intent.

### 2.2. Participants

The study population consisted of all females who died by suicide in Türkiye during the study period. A total of 12,868 cases were included in the analysis. Cases were categorised according to age group (<15, 15–24, 25–34, 35–44, 45–54, 55–64, 65–74, ≥75), marital status, and educational level. Age groups were defined according to the predefined classification used in the TURKSTAT suicide statistics database (<15, 15–24, 25–34, 35–44, 45–54, 55–64, 65–74, ≥75). These categories correspond to commonly used demographic age intervals representing different stages of the life course and were used in the present study to maintain consistency with the official national reporting system.

A total of 12,868 women died by suicide in Türkiye between 2009 and 2023. During this period, the suicide rate among women (per 100,000 population) ranged from 1.81 to 2.46, with the highest rate recorded in 2022 and the lowest in 2017. The annual number of suicides and corresponding rates are presented in Table 1. Of the women who died by suicide during this period, 6.4% (n = 824) were illiterate, 51.8% (n = 6668) were literate and/or had completed primary education, 14.0% (n = 1802) had completed secondary education or its equivalent, 15.6% (n = 2009) had completed high school or its equivalent, and 9.1% (n = 1171) were graduates of higher education institutions. The educational status of 3.1% (n = 394) was unknown. Regarding marital status, 39.2% (n = 5038) had never been married, 43.4% (n = 5585) were married, and 8.6% (n = 1107) were divorced. Additionally, 7.9% (n = 1023) were widowed, while the marital status of 115 women (0.9%) was not reported (Table 1).

### 2.3. Variables and Classification

Suicide reasons were classified into five categories based on TURKSTAT definitions:

1. Illness.
2. Family conflict.
3. Economic difficulties.
4. Emotional relationship issues and inability to marry the person of choice.
5. Other (including academic failure, business failure, and unknown or unspecified causes).

In the TURKSTAT database, some cases are classified as “unknown” or “unspecified” causes. Because these categories are conceptually similar and represent cases where a specific motive was not identified, they were grouped together under the “other” category in the present analysis. According to TURKSTAT definitions, the category ‘illness’ includes a broad range of health-related conditions reported during the death investigation process, encompassing both chronic physical diseases and mental health conditions. However, TURKSTAT does not provide sub-classification of the specific illness types, and therefore detailed clinical differentiation (e.g., psychiatric vs. somatic conditions) was not possible within this dataset.

The cause and method categories presented in this study reflect the original TURKSTAT classifications and were not modified by the authors. Because the dataset does not include ICD-10 codes for individual suicide cases, the five cause categories (illness, family conflict, economic hardship, emotional relationship issues, other) and the five method categories (hanging, toxic substances, jumping from height, firearms, other) cannot be mapped directly onto ICD-10 codes X60–X84. Another methodological consideration concerns the classification systems used for mortality statistics. Although suicide deaths have traditionally been coded according to the ICD-10 classification (codes X60–X84), the World Health Organization introduced the ICD-11 classification system on 1 January 2022. Because the publicly available TURKSTAT dataset used in this study does not provide individual ICD codes and relies instead on administrative categories, direct comparisons with ICD-11–based datasets may be limited in future epidemiological studies. Although suicide methods in international mortality statistics are typically classified according to ICD-10 codes X60–X84, the publicly available TURKSTAT dataset does not provide individual ICD-10 codes for each case. Instead, suicide methods are reported using predefined administrative categories such as hanging, ingestion of toxic substances, jumping from height, firearms, and other methods. These categories broadly correspond to commonly used ICD-10 suicide classifications but cannot be mapped directly to specific ICD-10 codes using the available dataset. As a result, ICD-10–based cross-national comparisons could not be performed.

Suicide methods were categorised into five groups:

1. Hanging
2. Ingestion of toxic substances
3. Jumping from a height
4. Use of firearms
5. Other (e.g., drowning, self-immolation, cutting, gas inhalation, and jumping in front of a vehicle).

### 2.4. Statistical Analysis

All statistical analyses were performed using SPSS software v27 (IBM Corp., Armonk, NY, USA). Descriptive statistics were used to summarise the data. Categorical variables such as education level, marital status, reason, and method of suicide were presented as frequencies (n) and percentages (%). Age-specific suicide rates per 100,000 population were calculated for each year using mid-year female population estimates provided by TURKSTAT. Crude suicide rates were reported because the primary aim of the study was to describe age-specific patterns of suicide within the national population rather than to perform international comparisons using age-standardised mortality rates. To examine the association between age groups and suicide reasons/methods, Pearson’s Chi-square test was applied. A *p*-value of less than 0.05 was considered statistically significant.

## 3. Results

Between 2009 and 2023, the causes of suicide among women in Türkiye showed significant variation across different age groups. Illness was the leading cause of suicide in most adult age categories, with its proportion increasing steadily with age. In this dataset, ‘illness’ reflects a broad administrative category that may include chronic physical conditions as well as mental health-related problems, although the specific illness types are not differentiated in TURKSTAT records. For instance, while 13.9% of women aged 15–24 died by suicide due to illness, this rate rose to 42.3% in the 55–64 age group. These categories represent administrative groupings provided by TURKSTAT and do not correspond to specific ICD-10 codes. Among females under the age of 15, illness (10.1%) and family conflict (10.5%) were the most common identifiable causes, although the majority of cases in this group were classified under “other” causes. In the 15–24 age group, besides illness, family conflict and emotional relationship issues also contributed notably to suicide cases. Economic hardship was the least common reason for suicide in younger age groups, especially among those under 35 years of age. In contrast, emotional relationship issues and the inability to marry the desired person were more prevalent in younger women but decreased significantly in older age brackets. The proportion of cases categorised under “other” causes remained consistently high across all age groups, especially among the youngest (<15 years) and middle-aged (35–54 years) women (Table 2).

In the older age groups, particularly among women aged 65–74 and 75 years and older, illness remained the predominant reason for suicide, accounting for 40.0% and 31.2% of cases respectively. This trend reinforces the association between aging and increased health-related vulnerabilities as a significant factor contributing to suicide. Conversely, family conflict, economic hardship, and emotional relationship issues were markedly less frequent in these older age groups. For instance, emotional relationship problems accounted for only 0.3% of suicides in the 65–74 age group and 0.7% in women aged 75 and older. Economic hardship was also relatively uncommon in these groups. In the category of women with unknown age, illness was again the leading cause (25.0%), followed by “other” causes (59.4%). Across all older age groups, the “other” category maintained a significant proportion, suggesting that non-specific or unreported motives remain a persistent challenge in understanding suicide causes among elderly women (Table 2).

An analysis of the methods of suicide among women in Türkiye from 2009 to 2023 reveals significant age-related differences. Across all age groups, hanging was the most frequently used method, especially among older women. For instance, the proportion of suicides by hanging was 35.8% in the 15–24 age group and increased progressively with age, reaching 63.5% among women aged 75 and older. The use of chemical substances (such as medications or toxic agents) was more commonly observed in younger and middle-aged women, particularly in the 15–34 age group, where it accounted for up to 12.4% of suicides. Jumping from a height was the second most prevalent method across nearly all age groups, maintaining a relatively stable rate of around 19–21%, with minimal variation. In contrast, use of firearms showed a marked decline with advancing age: while it accounted for 24.6% of suicides in girls under 15, it dropped to 2.0% in women aged 65–74 and to just 0.8% in those aged 75 and above. The “other” category—which included methods such as self-immolation, cutting, gas inhalation, and jumping in front of vehicles—remained consistently low across all age groups, most frequent in younger populations but never exceeding 10% in any group (Table 3).

There was a statistically significant difference in the causes of suicide among different age groups (*p* < 0.001). In all age categories, suicides due to “other” reasons were the most common. Among girls under the age of 15, 10.1% died by suicide due to illness, while this rate was 13.9% in the 15–24 age group, 24.6% in the 25–34 age group, 41.0% in the 45–54 age group, and 42.3% in the 55–64 age group. In the under-15 group, after “other” causes, the most frequent reasons for suicide were illness (10.1%) and family conflict (10.5%). Illness was also the most common cause of suicide after “other” reasons in the following age groups: 13.9% in 15–24, 24.6% in 25–34, 34.6% in 35–44, 41.0% in 45–54, 42.3% in 55–64, 40.0% in 65–74, 31.2% in those aged 75 and older, and 25.0% in individuals with unknown age. The least common cause of suicide across age groups was economic hardship among girls under 15 (0.8%), those aged 15–24 (1.0%), and 25–34 (2.6%). In the age groups of 35–44 (2.6%), 45–54 (1.0%), 55–64 (0.9%), 65–74 (0.3%), and 75+ (0.7%), the least frequent reason was emotional relationship issues or inability to marry by choice (Table 4).

**Figure 1:**
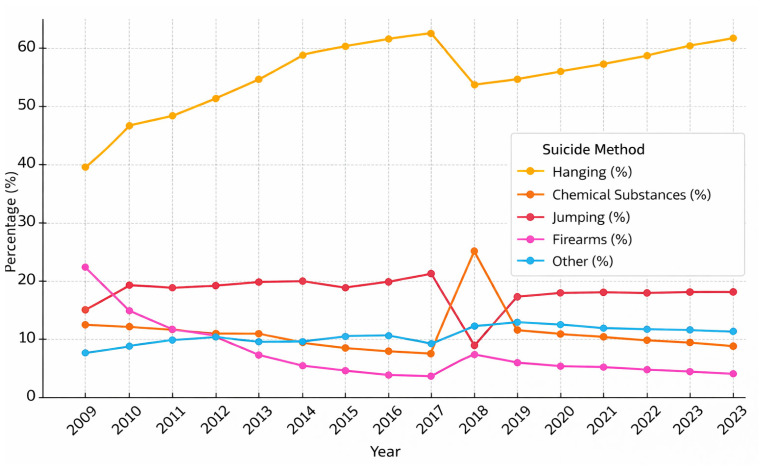
Distribution of Suicide Methods by Years (2009–2023).

**Figure 2:**
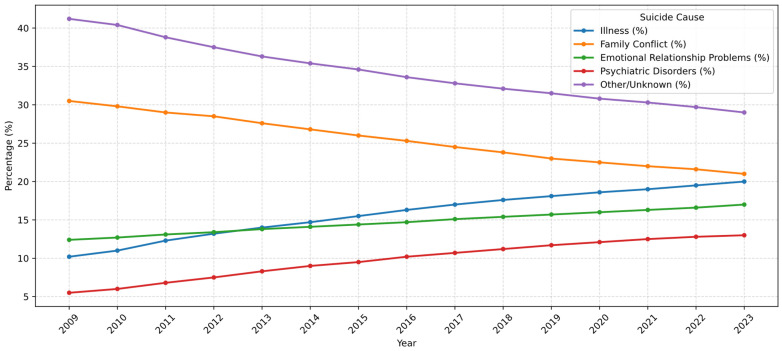
Distribution of Suicide Causes by Years (2009–2023).

When suicide methods were examined by age group, a statistically significant difference was observed among the groups (*p* < 0.001). In all age groups, hanging was the most commonly used method of suicide. Regarding the least common methods, the “other” category—which includes methods such as drowning, self-immolation, cutting, and gas inhalation—was most infrequently used among girls under 15 (6.3%), women aged 15–24 (6.9%), and those aged 25–34 (8.7%). Among individuals aged 35–44, the least preferred methods were both firearms and other methods, each accounting for 9.8% of cases. In the 45–54 (5.5%), 55–64 (2.5%), 65–74 (2.0%), and 75+ (0.8%) age groups, firearm use was the least observed method of suicide. Among those with unknown age, the least common methods were jumping from height and firearm use, each constituting 6.2% of suicides (Table 4, Figure 1 and Figure 2).

## 4. Discussion

This study presented one of the most comprehensive nationwide analyses to date of female suicides in Türkiye, spanning a 15-year period. Our findings reveal significant age-related differences in both the causes and methods of suicide among women. Notably, illness emerged as the most frequently reported reason for suicide across most adult age groups, while hanging was consistently the most common method regardless of age. These patterns reflect broader trends documented in national and international literature but also highlight gender- and culture-specific dynamics unique to Türkiye.

In addition to the age-related patterns observed in the present study, the annual distribution of suicide deaths presented in Figure 1 suggests several temporal fluctuations during the study period. In particular, a relative decrease around 2016–2017 followed by an increase towards 2021–2022 can be observed. Such turning points in suicide trends may reflect broader societal, economic, and public health influences. For instance, economic instability, social changes, and large-scale public health events such as the COVID-19 pandemic may have affected psychological well-being and suicide patterns in the population. However, because the present study was primarily designed to examine age-related differences in reported suicide reasons and methods rather than to formally model temporal trend changes, these observations should be interpreted cautiously. Future studies using time-series modelling approaches such as interrupted time-series or joinpoint regression could provide more detailed insights into temporal changes in suicide trends.

In our study, we found that chronic physical or psychiatric illnesses were the leading precipitating factors for suicide among women, particularly in middle and older adulthood. Waern et al. reported that serious physical illnesses were found to increase the risk of suicide in older individuals. In particular, conditions such as malignant diseases, neurological disorders, and vision loss were among the factors that independently increased the risk of suicide (Waern et al., 2002). Chan et al. reported that 60% of suicides aged 60 and older occurred in men, with depression being the most common diagnosis. It was also noted that suicides in older women were more frequently associated with a history of previous suicide attempts (Chan et al., 2024). Kilicarslan et al. emphasized that chronic disease burden, coupled with low access to mental health services, contributes significantly to female suicidality in Türkiye (Kilicarslan et al., 2023). Similarly, Kaya et al. showed that physical illness was a leading factor in suicide among elderly individuals, which aligns with our observation of increased illness-related suicides in the 55+ age groups (Kaya et al., 2020).

The predominance of hanging as the suicide method in all age groups is also notable. Although prior literature suggested that men typically choose more lethal means, such as firearms, while women more often opt for poisoning or overdose (Kartal et al., 2022), our study revealed a clear preference for hanging among Turkish women across all age groups. This finding may reflect access-related factors, cultural norms, or method familiarity. Akin and Konar also reported hanging as the most common method among both adolescent girls and boys in Türkiye, supporting the ubiquity of this method across age and gender (Akin & Konar, 2025). Atreya et al. conducted a study in Nepal analyzing completed suicides and found that hanging was the most frequently used method among both sexes, while illness and social pressure were among the primary contributing factors for female suicides, particularly in middle and older adulthood (Atreya et al., 2019). Although their study emphasized ambiguities in gender-based differences in method selection, our findings demonstrated a consistent age-related pattern, with hanging having become increasingly dominant with advancing age among Turkish women. Furthermore, similar to the Nepalese context, chronic physical or psychiatric conditions appeared as the principal drivers of suicide in our cohort, especially in women over the age of 45. These parallels highlighted the cross-cultural relevance of health-related vulnerabilities in female suicidality and emphasized the need for targeted public health interventions focusing on chronic disease management and emotional support for older women.

The low proportion of suicides attributed to economic hardship—especially among younger women—aligns with previous research suggesting that psychosocial and relational factors tend to be more influential suicide drivers for females, compared to men who are more affected by employment-related stressors (Han Yekdes et al., 2025). However, it is critical not to dismiss economic difficulty, as underreporting and misclassification under “other” or “unknown” causes may mask its true prevalence. Taktak has emphasized that suicides with unknown or vague causes represent a significant public health blind spot and are especially common in official Turkish statistics (Taktak, 2023). So et al. found that among young adults in Hong Kong, financial hardship indirectly contributed to increased suicidality through its impact on psychological distress, highlighting the complex interplay between economic factors and mental health (So et al., 2024). Additionally, Pathirathna et al. reported that financial crises due to loss of employment during the COVID-19 pandemic were associated with increased suicide attempts, underscoring the importance of accurately identifying and addressing economic stressors in suicide prevention efforts (Pathirathna et al., 2022).

Another factor that may have influenced suicide patterns during the study period is the COVID-19 pandemic. The pandemic introduced substantial social, economic, and psychological stressors worldwide, including social isolation, economic uncertainty, and disruptions in mental health services. Recent international studies examining suicide mortality during the first year of the pandemic across multiple European countries reported heterogeneous effects, with some countries showing temporary stabilization while others experienced increases in suicide rates, highlighting the complex relationship between pandemic-related stressors and suicidal behaviour (Lantos & Nyári, 2024). Although the present study was not designed to formally evaluate temporal trend changes associated with the pandemic, future research using time-series modelling techniques may provide further insights into the potential effects of COVID-19 on suicide patterns in Türkiye.

Another key finding is the relatively high prevalence of “other” causes across all age groups. This broad and ambiguous category, which includes academic failure, business loss, and unknown reasons, constituted more than half of all suicides in some subgroups. This trend is worrying, as it points to a potential deficiency in suicide reporting systems and limits the development of targeted prevention strategies. Comparable concerns have been raised by Kartal et al., who analysed 25 years of suicide data and criticized the high proportion of suicides with unexplained motives in Türkiye (Kartal et al., 2022).

International studies also highlight relevant parallels and contrasts. For example, Bertuccio et al., using global WHO data, reported a rise in youth suicides worldwide, with illness and interpersonal stressors dominating among girls (Bertuccio et al., 2024). Similarly, Arqueros et al. identified emotional trauma and relationship-related distress as major suicide risk factors in adolescents, findings that echo our results for women aged 15–34, where “emotional relationship issues and inability to marry by choice” were disproportionately represented (Arqueros et al., 2025).

In terms of methods, our finding that firearm use decreases significantly with age, reaching only 0.8% in women aged 75+, aligns with Han Yekdes et al., who noted that access to firearms is heavily regulated in Türkiye and tends to affect younger males more than older females (Han Yekdes et al., 2025). The consistently low use of chemical substances and jumping from height among older women may be attributed to physical limitations or accessibility barriers.

Taken together, our findings underscore the need for age- and gender-sensitive suicide prevention strategies in Türkiye. The observed age gradient in illness-related suicides calls for targeted screening and support programs for chronic disease patients, particularly older women. This aligns with recent studies emphasizing the importance of early identification and treatment of mental health issues among the elderly, as well as the provision of social support to mitigate suicide risk (Acoba, 2024; Mann et al., 2021; Sebti et al., 2024). The dominance of “other” as a reported cause suggests the urgent need to improve suicide classification systems and postmortem psychosocial investigations. Accurate identification of suicide motives is crucial for developing effective prevention strategies. Additionally, the persistence of emotional and relational motives in young women points to the importance of expanding school- and university-based mental health interventions. Programs such as the JED Campus initiative illustrate how nationwide, campus-based mental health strategies can be implemented to support student well-being and suicide prevention (MacPhee et al., 2021). Moreover, integrating resilience-building strategies into educational curricula has been shown to equip young individuals with coping mechanisms to handle stress and emotional challenges. These interventions, combined with efforts to promote gender equality and emotional resilience, are vital in addressing the unique mental health needs of young women.

Zeybek et al. examined suicide cases among individuals aged 65 and older in Türkiye between 2002 and 2019 and found that 74% of the cases were male and 26% female (Zeybek et al., 2023). Crude suicide rates in all older age groups were higher than those in the general population. Illness was identified as the most common cause of suicide, followed by marital conflict in women and financial difficulties in men. Hanging was the most frequently used method for both sexes, with a notable increase in firearm use over the years, particularly among men (Zeybek et al., 2023). Kaya et al. emphasized that physical illness was the leading cause of suicide among elderly individuals, particularly women, underlining the vulnerability of this demographic due to chronic disease and limited functional capacity (Kaya et al., 2020). Kartal et al., in their 25-year analysis of suicide data in Türkiye, also highlighted the elevated suicide rates in older populations and noted the persistent classification of many cases under ambiguous categories such as “other,” pointing to shortcomings in cause-of-death reporting systems (Kartal et al., 2022). Kilicarslan et al. further noted that among Turkish women, the presence of chronic illness combined with poor access to mental health services significantly increased suicidality (Kilicarslan et al., 2023). Together, these findings underscore the predominance of illness-related suicides among elderly women and reaffirm hanging as the most commonly employed suicide method in this group. The consistently low firearm use among older females may reflect cultural attitudes and accessibility limitations. In contrast, studies have shown that emotional and relationship-related issues are more prevalent among younger women, suggesting an age-related shift in suicide motivations. These observations highlight the importance of developing age- and gender-specific suicide prevention strategies in Türkiye.

Unlike many previous studies that focused either on general suicide trends or male-dominated datasets, this study provides a nationwide and gender-specific longitudinal analysis of female suicides in Türkiye over a 15-year period. While earlier research by Kartal et al. and Han Yekdes et al. evaluated national suicide patterns using aggregate data, they did not stratify findings by gender, age group, and method of suicide in an integrated manner (Han Yekdes et al., 2025; Kartal et al., 2022). Studies such as those by Akin and Konar or Kilicarslan et al. addressed specific age ranges or localized female populations but lacked a nationwide temporal scope. In contrast, our study distinguishes itself by combining age-stratified analysis with detailed cause- and method-specific trends, allowing for more targeted interpretation (Akin & Konar, 2025; Kilicarslan et al., 2023). Moreover, it sheds light on the persistent ambiguity in suicide causation—particularly the dominance of the “other” category—which has not been systematically explored in prior literature. By focusing solely on women, our research fills a notable gap and offers a critical gendered perspective necessary for designing effective prevention strategies and public health policies.

Beyond the comparison with previous studies, the present findings provide important insights into the potential prevention of suicidal behaviour among women. The observed temporal trends in suicide methods and underlying causes suggest that psychosocial stressors, emotional relationship problems, and psychiatric disorders may play an increasingly important role in female suicide patterns. These findings highlight the need for age-specific and women-focused suicide prevention strategies in Türkiye. In particular, strengthening mental health services, improving early detection of psychiatric disorders, expanding crisis intervention programs, and increasing social support mechanisms for women experiencing family or relationship-related stress may contribute to reducing suicide risk. Public health interventions focusing on awareness, access to mental health care, and early psychosocial support could therefore play a critical role in prevention efforts.

Another important issue concerns the reliability and completeness of suicide mortality records. Systems used to register suicide deaths may have several limitations both in Türkiye and internationally. The transition from ICD-10 to ICD-11 in international mortality statistics may also influence future cross-national comparisons of suicide data. Harmonization of administrative categories with updated ICD classifications may therefore improve the comparability of suicide research across countries. Underreporting may occur due to social stigma, cultural or religious factors, and the reluctance of families or authorities to classify deaths as suicide. In some cases, deaths may be recorded as accidents or deaths of undetermined intent, which may lead to an underestimation of the true burden of suicide. Differences in classification practices, reporting systems, and medico-legal procedures between countries can also affect the comparability of international statistics. Therefore, although national statistical databases provide valuable epidemiological information, the results should be interpreted with caution, considering the potential limitations of suicide reporting systems.

Earlier studies have examined suicide patterns in Türkiye from different perspectives, Asirdizer et al. analysed national suicide data between 1996 and 2005 and provided an early epidemiological overview of suicide trends in the country (Asirdizer et al., 2010). Goren et al. focused specifically on female suicides in Diyarbakir, highlighting regional patterns and sociocultural influences (Goren et al., 2004). More recently, Han Yekdeş et al. conducted a nationwide epidemiological analysis of suicide-related mortality between 2009 and 2022 (Han Yekdes et al., 2025). However, these studies either examined broader national suicide patterns or focused on specific regions or determinants. In contrast, the present study provides a detailed age-stratified analysis of reported suicide reasons and methods specifically among women across a 15-year nationwide dataset.

This study has some limitations. First, the data were obtained from the official records of the Turkish Statistical Institute, which rely on death certificates and administrative reporting. Therefore, misclassification or underreporting of suicide cases—particularly in conservative or rural regions—may have led to an underestimation of the true suicide burden. Additionally, the category ‘illness’ represents a broad administrative classification used by TURKSTAT and does not distinguish between physical illnesses and psychiatric conditions. Because detailed diagnostic information is not available at the individual level, illness-related suicides could not be further subclassified. Second, the category of “other causes,” which constituted a substantial proportion of cases across all age groups, limits the interpretability of specific psychosocial or cultural drivers of suicide. This ambiguity reflects a lack of nationwide psychological autopsy or detailed contextual investigation at the time of death. Third, the data lacked individual-level clinical information, such as psychiatric diagnoses, substance use history, or prior suicide attempts, which are known to be strong predictors of suicide but were unavailable in aggregate statistical data. Additionally, temporal factors such as seasonal variation or post-disaster effects (e.g., earthquakes, pandemics) were not accounted for in this analysis, although they may influence suicide rates. Lastly, this study focused exclusively on female suicides, and while it provides gender-specific insights, comparisons with male suicide patterns were beyond the scope of the current work. Additionally, the reported reasons for suicide in the TURKSTAT database are based on administrative records derived from police or gendarmerie investigation reports rather than detailed psychological autopsy data, which may limit the accuracy and clinical specificity of these classifications. Another methodological limitation concerns the operational definition used in national suicide statistics. In many mortality classification systems, deaths coded under ICD-10 categories X60–X84 are considered confirmed suicides, whereas deaths coded under categories of undetermined intent (e.g., Y20–Y34) may also include cases that are potentially related to suicide. Because the publicly available TURKSTAT dataset reports suicide deaths using predefined administrative categories rather than detailed ICD codes, it was not possible to evaluate deaths classified under undetermined intent categories. Therefore, the possibility that some suicide deaths may be underreported or misclassified cannot be entirely excluded. Future studies integrating qualitative analyses, psychological autopsies, and comparative gender evaluations could offer a more nuanced understanding of suicide dynamics in Türkiye.

## 5. Conclusions

In conclusion, this nationwide analysis demonstrates that female suicide in Türkiye is characterized by marked age-related differences in both reported reasons and methods. Illness-related suicides and hanging predominate in older age groups, whereas younger women exhibit a more heterogeneous pattern, with emotional and relationship-related factors and a wider range of methods being more prominent. The consistently high proportion of suicides classified under nonspecific categories highlights important limitations in current suicide reporting and diagnostic frameworks. These findings underscore the need for improved suicide classification systems, enhanced surveillance, and the implementation of age- and gender-sensitive prevention strategies that address the distinct risk profiles of women across the lifespan.

## Figures and Tables

**Table 1:** Annual number of female suicides and crude suicide rates per 100,000 population in Türkiye (2009–2023).

Year	Number of Female Suicides (n)	Crude Suicide Rate (per 100,000)
2009	787	2.19
2010	860	2.36
2011	801	2.17
2012	910	2.43
2013	870	2.29
2014	817	2.12
2015	888	2.28
2016	767	1.94
2017	723	1.81
2018	813	2.00
2019	792	1.92
2020	865	2.08
2021	931	2.22
2022	1045	2.46
2023	999	2.34

**Table 2:** Distribution of female suicides by age group and cause of suicide in Türkiye (2009–2023).

Age Group (n)	Illness n (%)	Family Conflict n (%)	Economic Hardship n (%)	Emotional Relationship n (%)	Other n (%)
<15 (n = 666)	67 (10.1)	70 (10.5)	5 (0.8)	12 (1.8)	512 (76.9)
15–24 (n = 3965)	551 (13.9)	412 (10.4)	40 (1.0)	190 (4.8)	2772 (69.9)
25–34 (n = 2403)	591 (24.6)	233 (9.7)	63 (2.6)	96 (4.0)	1420 (59.1)
35–44 (n = 1870)	647 (34.6)	108 (5.8)	50 (2.7)	49 (2.6)	1016 (54.4)
45–54 (n = 1409)	578 (41.0)	63 (4.5)	25 (1.8)	14 (1.0)	729 (51.7)
55–64 (n = 1073)	454 (42.3)	32 (3.0)	21 (2.0)	10 (0.9)	556 (51.8)
65–74 (n = 738)	295 (40.0)	12 (1.6)	9 (1.2)	2 (0.3)	420 (56.9)
75+ (n = 712)	222 (31.2)	11 (1.5)	6 (0.8)	5 (0.7)	468 (65.7)

**Table 3:** Distribution of Female Suicides in Türkiye by Age and Method of Suicide (2009–2023).

Age Group (n)	Hanging n (%)	Chemical Substances n (%)	Jumping from Height n (%)	Firearms n (%)	Other Methods n (%)
<15 (n = 666)	301 (45.2)	50 (7.5)	109 (16.4)	164 (24.6)	42 (6.3)
15–24 (n = 3965)	1421 (35.8)	490 (12.4)	807 (20.4)	973 (24.5)	274 (6.9)
25–34 (n = 2403)	1143 (47.6)	257 (10.7)	473 (19.7)	321 (13.4)	209 (8.7)
35–44 (n = 1870)	947 (50.6)	193 (10.3)	364 (19.5)	183 (9.8)	183 (9.8)
45–54 (n = 1409)	776 (55.1)	147 (10.4)	280 (19.9)	78 (5.5)	128 (9.1)
55–64 (n = 1073)	644 (60.0)	86 (8.0)	219 (20.4)	27 (2.5)	97 (9.0)
65–74 (n = 738)	456 (61.8)	53 (7.2)	140 (19.0)	15 (2.0)	74 (10.0)
75+ (n = 712)	452 (63.5)	41 (5.8)	153 (21.5)	6 (0.8)	60 (8.4)
Unknown (n = 32)	16 (50.0)	8 (25.0)	2 (6.2)	2 (6.2)	4 (12.5)

**Table 4:** Distribution of Suicide Causes and Methods Among Women by Age Group in Türkiye (2009–2023).

**Causes of Suicide**					
**Age Group (n)**	**Illness** **n (%)**	**Family** **Conflict n (%)**	**Economic** **Hardship n (%)**	**Emotional** **Relationship n (%)**	**Other n (%)**
<15 (n = 666)	67 (10.1)	70 (10.5)	5 (0.8)	12 (1.8)	512 (76.9)
15–24 (n = 3965)	551 (13.9)	412 (10.4)	40 (1.0)	190 (4.8)	2772 (69.9)
25–34 (n = 2403)	591 (24.6)	233 (9.7)	63 (2.6)	96 (4.0)	1420 (59.1)
35–44 (n = 1870)	647 (34.6)	108 (5.8)	50 (2.7)	49 (2.6)	1016 (54.4)
45–54 (n = 1409)	578 (41.0)	63 (4.5)	25 (1.8)	14 (1.0)	729 (51.7)
55–64 (n = 1073)	454 (42.3)	32 (3.0)	21 (2.0)	10 (0.9)	556 (51.8)
65–74 (n = 738)	295 (40.0)	12 (1.6)	9 (1.2)	2 (0.3)	420 (56.9)
75+ (n = 712)	222 (31.2)	11 (1.5)	6 (0.8)	5 (0.7)	468 (65.7)
**Methods of Suicide**					
**Age Group (n)**	**Hanging** **n (%)**	**Chemical** **Substances n (%)**	**Jumping from** **Height n (%)**	**Firearms n (%)**	**Other n (%)**
<15 (n = 666)	301 (45.2)	50 (7.5)	109 (16.4)	164 (24.6)	42 (6.3)
15–24 (n = 3965)	1421 (35.8)	490 (12.4)	807 (20.4)	973 (24.5)	274 (6.9)
25–34 (n = 2403)	1143 (47.6)	257 (10.7)	473 (19.7)	321 (13.4)	209 (8.7)
35–44 (n = 1870)	947 (50.6)	193 (10.3)	364 (19.5)	183 (9.8)	183 (9.8)
45–54 (n = 1409)	776 (55.1)	147 (10.4)	280 (19.9)	78 (5.5)	128 (9.1)
55–64 (n = 1073)	644 (60.0)	86 (8.0)	219 (20.4)	27 (2.5)	97 (9.0)
65–74 (n = 738)	456 (61.8)	53 (7.2)	140 (19.0)	15 (2.0)	74 (10.0)
75+ (n = 712)	452 (63.5)	41 (5.8)	153 (21.5)	6 (0.8)	60 (8.4)

## Data Availability

The data supporting the findings of this study are available from the corresponding author upon reasonable request.

## Abbreviations

ICD-10	International Classification of Diseases, 10th Revision
ICD-11	International Classification of Diseases, 11th Revision
JED	The Jed Foundation (JED Campus Program)
SPSS	Statistical Package for the Social Sciences
TURKSTAT	Turkish Statistical Institute
WHO	World Health Organization
